# Stratification of Patients with Burning Mouth Syndrome in the Croatian Population: A Single-Center Cross-Sectional Study

**DOI:** 10.3390/neurosci6020033

**Published:** 2025-04-14

**Authors:** Ana Glavina, Ana Trlaja, Dinko Martinović, Antonija Tadin, Liborija Lugović-Mihić

**Affiliations:** 1 Department of Dental Medicine, University Hospital of Split, 21000 Split, Croatia; 2 Department of Oral Medicine, Study of Dental Medicine, School of Medicine, University of Split, 21000 Split, Croatia; 3 Study of Dental Medicine, School of Medicine, University of Split, 21000 Split, Croatia; ana.trlaja98@gmail.com; 4 Department of Maxillofacial Surgery, University Hospital of Split, 21000 Split, Croatia; d.m.993@hotmail.com; 5 Department of Pathophysiology, School of Medicine, University of Split, 21000 Split, Croatia; 6 Department of Restorative Dental Medicine and Endodontics, Study of Dental Medicine, School of Medicine, University of Split, 21000 Spit, Croatia; dr.antonija.tadin@gmail.com; 7 Department of Dermatovenereology, University Hospital Center Sestre Milosrdnice, 10000 Zagreb, Croatia; liborija@gmail.com; 8 School of Dental Medicine, University of Zagreb, 10000 Zagreb, Croatia

**Keywords:** burning mouth syndrome, xerostomia, dysgeusia, glossopyrosis, glossodynia

## Abstract

The objective of the study was to determine the relationship between burning, xerostomia, dysgeusia and other subjective symptoms in patients with burning mouth syndrome (BMS). This cross-sectional study was conducted at the Dental Polyclinic Split, Split, Croatia. A total of 71 patients with BMS, i.e., 60 women and 11 men, were included in the study. The patients were divided into four subgroups: burning (B), burning and xerostomia (BX), burning and dysgeusia (BD), burning, xerostomia and dysgeusia (BXD). The following data were collected from all patients: sociodemographic status, comorbidities, medications, characteristics of the burning, presence of other subjective symptoms, topography of the burning. The majority of patients with BMS were women (86.0%) with an average age of about 65 years. Gastrointestinal diseases were the most common comorbidity (48.35%), and the most commonly used medications were proton pump inhibitors (PPIs) (29.8%). In the largest number of patients (N = 34), the burning symptom worsened in the evening hours (*p* = 0.059). The majority of BMS patients suffered from burning symptoms that occurred continuously (N = 54, 75.13%) and from an improvement (reduction/cessation) of symptoms during meals (N = 54, 76.65%). Of the other subjective symptoms, changes in the morphology of the tongue (10.6%) and a feeling of swelling (9.1%) were the most common. The tongue was the most common localization (67.35%). The multivariable logistic regression analysis showed a statistically significant effect of female gender (*p* = 0.049) as a potential positive predictor in subgroup B. The sociodemographic and medical data collected cannot explain the different occurrence of symptoms in the four subgroups of patients with BMS.

## 1. Introduction

Burning mouth syndrome (BMS) is a complex chronic pain disorder characterized by spontaneous unpleasant sensations (pain, burning) of the clinically healthy oral mucosa, excluding all local and systemic causes [1]. It is characterized by a strict oral localization of symptoms. The first descriptions of burning mouth date back to the mid-19th century, while BMS was first described in the literature in 1935. The name glossodynia was given by Butlin and Oppenheim. Until BMS was described in 1990, this entity was referred to by numerous other terms, e.g., glossopirosis, stomatopirosis, glossodynia, stomatodynia and oral dysesthesia. All these terms were combined under the name BMS in 1990 [2]. According to the International Classification of Orofacial Pain 2020, BMS is defined as “intraoral burning or dysesthesia lasting more than two hours per day and longer than three months, without visible causative lesions on clinical examination and laboratory findings” [3].

The etiology is multifactorial, and possible etiologic factors include: local, systemic, psychological, neurologic, and idiopathic [4]. BMS is a diagnostic and therapeutic challenge, even for an experienced clinician. It is a diagnosis of exclusion, which means that BMS requires a detailed history, a clinical-oral examination and certain diagnostic procedures and laboratory tests.

The incidence and prevalence of BMS varies, so epidemiologic data should be interpreted with caution. True epidemiologic data are difficult to obtain because there are no universally accepted diagnostic criteria, the age of the population studied varies, and a variety of local and systemic factors can cause burning mouth symptoms, which may lead to an overestimation of the prevalence and low awareness of this syndrome among doctors of dental medicine and primary care physicians. The prevalence of BMS in the world is unknown as almost all studies were conducted in European and North American populations and different diagnostic criteria were used in the different studies [5]. It is estimated that 4.0% of the general population is affected by BMS [6,7,8,9,10]. It occurs in people aged between 27 and 87 years. The incidence of BMS increases with age in both men and women and is highest in women between the ages of 60 and 69 [11].

BMS patients experience and describe unpleasant oral sensations differently, from mild irritation to severe pain/burning. The most common sensory symptoms are burning, pain, hyperalgesia, dysesthesia and xerostomia (subjective sensation of dry mouth) [12]. However, burning is the most common symptom in BMS patients [13]. The localization of the burning is not pathognomonic, although the anterior 2/3 of the tongue, the lateral sides of the tongue, the hard palate and the lower lip are most commonly affected [2,14]. The burning can affect the whole oral cavity. It is spontaneous, independent of the anatomical distribution of the nerves, bilaterally symmetrical, of moderate to severe intensity, daily with gradual worsening towards evening and persists for years with periods of remission. The burning symptoms rarely occur at night. A typical and diagnostically significant feature is the reduction/ceasing of symptoms when eating and drinking, chewing gum and eating sweets. Some patients report that the burning occurs, worsens and intensifies when certain foods (hot, spicy, acidic, carbonated and alcoholic beverages) are consumed [15]. More than 70.0% of BMS patients have a diminished (hypogeusia) and altered taste (dysgeusia), usually bitter and/or metallic [16]. About 2/3 of BMS patients suffer from xerostomia and also report a sensation of sand in the mouth [17]. The burning symptom can occur in isolation or in combination with dysgeusia and/or xerostomia.

The objective of this study was to determine the relationship between burning, xerostomia, dysgeusia and other subjective symptoms (e.g., subjective feeling of swelling) in four subgroups (burning; burning and xerostomia; burning and dysgeusia; burning, xerostomia and dysgeusia) of BMS patients. The specific aim of the study was to further characterise BMS patients in the Croatian population. The hypothesis was that four subgroups of BMS patients would have the same potential positive predictors of burning, xerostomia and dysgeusia.

## 2. Materials and Methods

### 2.1. Study Design, Subjects, Inclusion and Exclusion Criteria

This cross-sectional study was conducted at the Dental Polyclinic Split, teaching base of the School of Medicine, University of Split (study of Dental Medicine), Split, Croatia. The study included BMS patients in the period from June 2019 to July 2024. The study was approved by the Ethics Committee of the School of Medicine, University of Split, Split, Croatia (Class: 029-01/24-02/0001, Reg. No.: 2181-198-03-04-24-0043) on 29 April 2024. To ensure quality and transparency, the guidelines of the STROBE statement for cross-sectional studies were followed (Table S1) [18]. The study protocol was explained to each subject (verbally and in writing), and after signing the informed consent form, they were enrolled in the study. All subjects who did not understand the nature and purpose of the study and the content of the informed consent form were excluded from the study. The study was conducted in accordance with the principles of the Declaration of Helsinki (1964) and its subsequent amendments.

A total of 71 subjects were included in the study, 60 women and 11 men. The age ranged from 31 to 86 years. The subjects were divided into four subgroups:

1. The first subgroup consisted of patients whose main symptom was burning (B);
2. The second subgroup consisted of patients with symptoms of burning and xerostomia (BX);
3. The third subgroup consisted of patients with symptoms of burning and dysgeusia (BD);
4. The fourth subgroup consisted of patients with symptoms of burning, xerostomia and dysgeusia (BXD).

The inclusion criterion was:

1. Patients who met the diagnostic criteria for the diagnosis of BMS according to the International Classification of Orofacial Pain, 1st edition [3].

The exclusion criteria were:

1. Patients who had pathologic changes in the oral mucosa;
2. Hyposalivation;
3. Patients who had received certain groups of medications for hypertension (ACE inhibitors), corticosteroids, antibiotics, antineoplastic, neurological and psychoactive therapies in the last year;
4. Smokers and alcoholics;
5. Patients who have received radiotherapy to the head and neck area;
6. Patients who have been diagnosed with Sjögren’s disease (SjD);
7. Underage patients (<18 years).

### 2.2. Clinical Evaluation

The same oral medicine specialist (A.G.) with five years of specialist experience took the medical history (sociodemographic status, symptom duration (months), comorbidities, medications) and performed the clinical-oral examination. The following parameters were recorded in all subjects: complete blood count (CBC), blood glucose level, serum iron (Fe), unsaturated iron binding capacity (UIBC), total iron binding capacity (TIBC), ferritin, folic acid, vitamin B12, in order to exclude their systemic influence on the oral cavity.

The level of education was recorded as primary school, high school or faculty degree. Occupational status was recorded as unemployed, employed or retired. The characteristics of the occurrence of burning were recorded as follows: worst in the morning, worst in the evening, same morning/evening, continuous, improvement during eating. In addition to the presence of burning, xerostomia and dysgeusia, other subjective symptoms were also recorded in the BMS patients [*globus hystericus* (sensation of a lump/mass in the throat), paresthesia (tingling), dysesthesia (disturbed/spontaneous sensation without external or internal stimuli), feeling of swelling, feeling of hypersalivation, change in tongue morphology)]. The burning topography was recorded at the following sites in the oral cavity: generalized, gingiva, lips, cheeks, tongue and palate.

### 2.3. Salivary Flow Rate (SFR)

All subjects underwent a sialometry test according to the guidelines of Navazesh M et al. for the collection of unstimulated whole saliva (UWS) and stimulated whole saliva (SWS) [19]. Prior to saliva collection, all subjects gargled their oral cavity with water to avoid contamination from other sources. Subjects collected UWS using the “spit method” while resting for five minutes at least one hour after eating. Salivary flow was then stimulated with a 1.0% solution of vitamin C (1 g ascorbic acid in 1 dcl water) and SWS was collected for five minutes [20]. Subjects were diagnosed with hyposalivation if the values of UWS were ≤0.1 mL/min and SWS ≤ 0.5 mL/min, and they were excluded from the study [21]. This excludes one of the local factors in BMS patients.

### 2.4. Instruments

#### Visual Analogue Scale (VAS)

The intensity of the burn/pain symptoms was measured using the VAS. This is a 100 mm measuring scale on which the numbers are indicated from zero to ten. Zero means no burning/pain and ten means the worst possible burning/pain.

### 2.5. Statistical Analysis

Statistical analysis of the data was performed using the statistical program MedCalc for a personal computer (Medcal Software, Ostend, Belgium, version 23.0.2). The Kolmogorov-Smirnov test was used to assess the normality of the distribution. The qualitative data are presented as whole numbers and percentages, the quantitative data (depending on the normality of the distribution) either as arithmetic mean (AM) ± standard deviation (SD) or as median (M) (interquartile range, IQR). The comparison between four groups of normally distributed quantitative variables was carried out using one-way analysis of variance (ANOVA) with a *post hoc* Scheffe test. The comparison between qualitative variables was carried out using the chi-square test. In addition, a multivariable logistic regression analysis of independent predictors was performed for four subgroups of BMS patients and the odds ratio (OR), 95.0% confidence interval (CI) and *p*-value were reported. The level of statistical significance was set at *p* < 0.05.

## 3. Results

### 3.1. Clinical Data and Sociodemographic Status

A total of 71 subjects were included in the study, 60 women and 11 men. Most patients were in the BXD group (N = 23, 32.4%), the fewest in the BD subgroup (N = 14, 19.7%). The majority of subjects in all four subgroups of BMS patients were women with a mean age of 65.2 years. There was no statistically significant difference between the subgroups in terms of gender and age (*p* = 0.310, *p* = 0.272). In all four subgroups, the majority of patients had a high school degree (N = 46, 64.8%; *p* = 0.191). Patients in subgroups B (N = 19, 26.8%), BX (N = 15, 21.1%) and BXD (N = 23, 32.4%) were predominantly retired, while patients in subgroup BD (N = 14, 19.7%) were predominantly employed (*p* = 0.146, *p* = 0.073) (Table 1).

### 3.2. Comorbidities and Medications

Gastrointestinal diseases were the most common comorbidity in all four subgroups of BMS patients (N = 35, *p* = 0.537). The second most common comorbidity in all four subgroups of BMS patients was hypertension (N = 28, *p* = 0.915) and the third most common was hypothyroidism (N = 10, *p* = 0.724). None of the comorbidities showed a statistically significant difference between the four subgroups of BMS patients (Table 2).

The most frequently used medications were proton pump inhibitors (PPIs) (N = 22, *p* = 0.280). This was followed by calcium channel blockers (CCBs) (N = 20, *p* = 0.981) and analgesics (N = 15, *p* = 0.196). Of all medications, PPIs were most frequently used in subgroups B (47.4%) and BXD (30.4%). The BX subgroup used analgesics most frequently (40.0%), the BD subgroup CCBs (28.6%). There were no statistically significant differences in medication use between all four subgroups of BMS patients (Table 3).

### 3.3. Symptom Characteristics

In the largest number of patients (N = 34), the symptoms worsened towards the evening (*p* = 0.059). Looking at the subgroups, the majority of patients in subgroups B (N = 8, 42.1%) and BX (N = 8, 53.3%) had the same intensity of symptoms in the morning and evening (*p* = 0.281). The majority of patients in subgroups BD (N = 10, 71.4%) and BXD (N = 13, 56.5%) had a worsening of symptoms towards the evening (*p* = 0.059). The majority of patients in subgroups B, BX and BXD had continuous burning symptoms (N = 17, 89.5%; N = 12, 80.0%; N = 17, 73.9%; *p* = 0.185). The majority of patients in all four subgroups of BMS patients (B, BX, BD, BXD) reported that they experienced an improvement, i.e., a reduction or cessation of burning symptoms, when eating (*p* = 0.826) (Table 4).

In addition to burning, xerostomia and dysgeusia, the most common subjective symptoms experienced by BMS patients were a change in tongue morphology (*p* = 0.727) and a feeling of swelling in various areas of the oral cavity (*p* = 0.540). Patients in the BXD subgroup had the longest symptom duration (21.8 ± 32.9 months), i.e., the time from the first onset of symptoms to presentation to the doctor of dental medicine and final diagnosis of BMS. Patients in subgroup BD had the shortest symptom duration (9.1 ± 10.1 months; *p* = 0.557). Patients in subgroup B had a lower intensity of burning symptoms (5.3 ± 1.8) compared to patients in subgroups BX, BD and BXD (*p* = 0.216) (Table 5).

### 3.4. Topography

The most frequent localization of burning symptoms in all subgroups of BMS patients was the tongue (N = 48, 67.6%; *p* = 0.675). The second most common localization was the lip in subgroups B (N = 4, 21.1%) and BD (N = 4, 28.6%) and the palate in subgroups BX (N = 5, 33.3%) and BXD (N = 7, 30.4%). Generalized involvement of the oral cavity was equally frequent and most pronounced in subgroups B and BD. There were no statistically significant differences in burn topography between the four subgroups of BMS patients (Table 6).

### 3.5. Regression Analysis

A multivariable logistic regression analysis of independent predictors (age, female gender, worst in the morning, worst in the evening, same morning/evening, symptom duration, VAS) was performed to identify possible predictors of burning, xerostomia and dysgeusia in four subgroups of BMS patients (B, BX, BD, BXD). There was a statistically significant effect of female gender as a potential positive predictor in subgroup B (*p* = 0.049) (Table 7).

Multivariable logistic regression analysis revealed no statistically significant influence of age, female gender, worst in the morning, worst in the evening, same morning/evening, symptom duration and VAS as potential positive predictors in subgroups BX, BD and BXD (Table 8, Table 9 and Table 10).

## 4. Discussion

The hypothesis was not confirmed by the results, i.e., we could not prove that the symptoms (burning, xerostomia, dysgeusia) have the same potential positive predictors in the four subgroups of BMS patients. The regression analysis showed a statistically significant effect of female gender as a potential positive predictor only in subgroup B. The objective of our study was to stratify BMS patients in the Croatian population by examining sociodemographic data, comorbidities and medication, characteristics of burning, presence of other subjective symptoms and topography of burning. A total of 71 patients with BMS from one center in Croatia, the city of Split, were included in the study. The literature search revealed that this is the first comprehensive study of BMS patients in Croatia. When comparing the results with other studies conducted worldwide, similarities emerge.

BMS is a disease with many unknowns, from etiology to therapy. It is a syndrome with numerous symptoms. The burning sensation in the oral cavity itself rarely occurs in isolation and is usually associated with other symptoms (such as xerostomia and/or dysgeusia), making diagnosis difficult for physicians/specialists [22].

It is known that certain groups of medications such as antipsychotics, tricyclic antidepressants (TCAs), benzodiazepines, antihypertensives (ACE inhibitors), bronchodilators, statins, chemotherapy and radiotherapy in the head and neck region can lead to BMS. Diseases and conditions such as SjD, systemic lupus erythematosus (SLE), rheumatoid arthritis (RA), diabetes (DM), Parkinson’s disease, anorexia, bulimia, psychogenic disorders (stress, anxiety, depression) and smoking also lead to BMS [23]. Therefore, the above-mentioned factors were exclusion criteria in this study, which aimed to gain new insights into the syndrome.

The study most similar to ours was conducted in Italy at the University of Naples Federico II from March 2019 to February 2022. It was a single-centre cross-sectional study with 500 BMS patients [24]. Many of the results of this study are consistent with ours. Numerous studies have found a higher prevalence of the syndrome in women than in men [22,23,24]. In our study, women were more frequently affected than men (86.0%), compared with 73.6% in the Italian study. The syndrome occurs on average in the sixth decade of life [23,24]. This was also shown by the results of this study in all four subgroups of BMS patients.

Gastrointestinal diseases were the most common comorbidity in all four subgroups of BMS patients. Although they were the most common, it is known that they are not the cause of the syndrome, but only a comorbidity [23]. Hypertension was the second most common comorbidity, hypothyroidism the third most common. In the Italian study, the order of the most common comorbidities was different. The most common was hypertension, then hypercholesterolemia and finally gastrointestinal diseases [24]. Spanish researchers concluded that thyroid alterations (especially hypothyroidism) can lead to secondary BMS [25]. Our findings are partially consistent with the results of the Italian and Spanish studies.

In our study, the most commonly used medications were PPIs. These are drugs that reduce gastric acid secretion and are the most common indication for gastroesophageal reflux disease (GERD) and reflux esophagitis [26]. Considering that gastrointestinal diseases were the most common comorbidities in this study, it is understandable that PPIs are the most commonly used medications. One of the side effects of these drugs is xerostomia. PPIs are used in the BX (N = 3, 20.0%) and BXD (N = 7, 30.4%) subgroups, in which one of the subjective symptoms is xerostomia. They should be prescribed more rationally [27]. This is also supported by the fact that PPIs are among the most frequently prescribed medications. However, their indication is questionable or inappropriate in 25.0% to 70.0% of cases [28]. In addition, the frequent use of PPIs leads to dysgeusia [29]. Thus, 21.4% (N = 3) of BD and 30.4% (N = 7) of BXD patients with dysgeusia symptoms took PPIs, which is another reason for more responsible prescribing of these medications. It is always recommended to consult a gastroenterologist to clarify the correct indication and rational use of PPIs in BMS patients with subjective symptoms of xerostomia and dysgeusia. In this study, PPIs followed CCBs and analgesics in terms of frequency of use. In an Italian study, antiplatelets, statins and PPIs were the most frequently used medications in BMS patients [24]. In a study conducted in Taiwan, psychotropic medications were the most commonly used, followed by PPIs and antihypertensives [30]. Brazilian researches came to similar conclusions. The most commonly used medications were antihypertensives, benzodiazepines and antidepressants [31]. Examination of the global results (excluding medications that were considered exclusion criteria in our study) suggests that BMS patients mostly use similar medications, i.e., PPIs, antihypertensives and statins.

With regard to the characteristics of burning, this study showed that the intensity of symptoms worsened towards the evening in most subjects (N = 34, 47.6%). In 36.8% (N = 26) of the BMS patients, the intensity was the same in the morning/evening. In the majority of BMS patients, the burning symptoms occurred continuously (N = 54, 75.1%) and improved (reduced/ceased) during meals (N = 54, 76.7%). In the Italian study, most patients (N = 271, 54.7%) had the same intensity of symptoms in the morning/evening. In the majority of Italian BMS patients, the burning symptoms occurred continuously (N = 307, 61.7%). Improvement during meals (N = 123, 28.4%) occurred three times less frequently in Italian BMS patients than in ours [24]. The results of a study conducted in Minnesota, a state in the United States of America, showed that symptoms were continuous in 86.6% of BMS patients [7]. This suggests that burning symptoms are continuous in BMS patients in Europe (based on available studies in European countries) and North America.

All BMS patients (N = 71) in this study had the burning. Only 19 BMS patients reported burning as the main symptom, without xerostomia and dysgeusia. In this study, xerostomia was present in 38 BMS patients (50.0%) and dysgeusia in 37 (50.0%). In a study from Brazil, 54.8% of patients had xerostomia and 32.2% had dysgeusia [31]. This indicates that burning rarely occurs in isolation, but is more often associated with other symptoms. In this study, in addition to the main symptoms (burning, xerostomia, dysgeusia), BMS patients most frequently experienced other subjective symptoms: a change in tongue morphology and a feeling of swelling in various areas of the oral cavity. In an Italian study, in addition to the main symptoms, *globus hystericus*, a change in tongue morphology and the sensation of a foreign body in the mouth were the most common [24]. In another Italian study, which was also conducted in Naples in 2021, pain, *globus hystericus* and dysmorphophobia were mentioned as other subjective symptoms [32].

The BMS patients rated the intensity of the burn/pain using the VAS. In this study, patients in subgroup B had the lowest mean VAS score (5.3 ± 1.8) compared to the other three subgroups of BMS patients. It is evident that the intensity of burning/pain is higher when xerostomia and/or dysgeusia are present. In a study from Minnesota, it was found that mild intensity of symptoms occurred in 41.6% of cases [7]. This suggests that BMS patients usually have mild/moderate intensity of symptoms. This facilitates the therapeutic approach for doctors of dental medicine/oral medicine specialists. The therapeutic approach should be compassionate and empathetic, providing information about the BMS symptoms and the limitations of treatment. It is important to explain to the patient that this is not a life-threatening condition/disease. This reduces anxiety and leads to psychological relief and consequently a reduction in the intensity of burn/pain symptoms [33,34,35]. The BXD subgroup had the longest symptom duration to diagnosis of BMS, while the BD subgroup had the shortest. This suggests that the greater number of symptoms makes the timely diagnosis of BMS more difficult.

In this study, the tongue was the most common localization of burning symptoms in 67.4% (N = 48) of BMS patients. It was predominant in all four subgroups of BMS patients. The lips (N = 17, 24.0%) were the second most common topographical location, and the palate was third (N = 16, 22.6%). The same order of symptom topography was found in an Italian study, in which the tongue was affected in 91.1% of BMS patients, the lips in 62.2% and the palate in 61.0% [24]. A study from Spain found that the most common localization was also the tongue (66.7%), 40.0% of which was at the tip of the tongue. The second most common site was the palate (26.7%) and the third most common was the lips (16.7%) [36]. These results are consistent with the results of our study. Thus, from these three European (Mediterranean) studies, we can philtre out the tongue, lips and palate as the most common localizations of symptoms in BMS patients. In a study from Minnesota, the tongue was affected in 81.9% of BMS patients [7]. This is another source that contributes to the tongue being the most common topographical site of burning in BMS patients in Europe and North America.

The advantage of this study is that it is the first comprehensive investigation of this clinical entity in Croatia. In addition, we consider the exclusion of ACE inhibitors in BMS patients as another advantage of this study (as other studies in the discussion did not do this), which may have biased the results. However, this study also has some disadvantages. First, only a relatively small number of patients from a single-center participated. The small number of male participants and the age homogeneity limit the ability to generalize gender-specific conclusions. Secondly, this is a cross-sectional study. Thirdly, it is possible that certain information was overlooked when taking the medical history (e.g., other subjective symptoms), which impaired obtaining a complete clinical picture of BMS. However, these limitations do not affect the main objective of this study, which was to confirm known epidemiologic trends and gain additional insight into the characteristics of BMS in the most affected population. Furthermore, given the complexity of BMS, the limitations of regression analysis should be taken into account. Further multicenter longitudinal studies in Croatia are needed to clarify the clinical picture and the complex etiopathogenetic mechanism of the syndrome.

## 5. Conclusions

This study showed that most BMS patients were women in their sixties. Most BMS patients had a high school degree and were retired. Gastrointestinal diseases were the most common comorbidity, and PPIs were the most commonly used medications. In most BMS patients, the intensity of burning symptoms worsened towards evening. The burning was continuous and the symptoms improved (reduced/ceased) during meals. Burning rarely occurred in isolation, but mostly in combination with xerostomia and dysgeusia. The most common other subjective symptoms were a change in tongue morphology and a feeling of swelling in various parts of the oral cavity. The tongue was the most common site for burning symptoms. BMS is accompanied by a broad spectrum of symptoms and other subjective complaints that often overlap. As there are no universally accepted diagnostic criteria and objective measurement tools, careful evaluation of objective and subjective symptoms is required.

## Figures and Tables

**Table 1:** Comparison of sociodemographic data between four subgroups of BMS patients.

Parameter	B N = 19	BX N = 15	BD N = 14	BXD N = 23	*p* *
Gender (N, %)					
Man	5 (26.3)	2 (6.7)	2 (14.3)	2 (8.7)	0.310 *
Woman	14 (73.7)	13 (93.3)	12 (85.7)	21 (91.3)	
Age (years)	65.0 ± 11.9	63.7 ± 16.1	62.2 ± 10.2	69.9 ± 12.1	0.272 ^†^
Education (N, %)					
Primary school	3 (15.8)	4 (26.7)	2 (14.3)	7 (30.4)	0.570 *
High school	14 (73.7)	10 (66.7)	11 (78.6)	11 (47.8)	0.191 *
Faculty	2 (10.5)	0	1 (7.1)	4 (17.4)	0.356 *
Employment (N, %)					
Unemployed	0	2 (13.3)	0	1 (4.3)	0.209 *
Employed	8 (42.1)	5 (33.3)	9 (64.3)	5 (21.7)	0.073 *
Retired	11 (57.9)	8 (53.3)	5 (35.7)	17 (73.9)	0.146 *

All data are given as whole numbers (percentage) or arithmetic mean ± standard deviation. * chi-square test. † One-way analysis of variance (ANOVA) with post hoc Scheffe test. Abbreviations: BMS—burning mouth syndrome; B—burning; BX—burning, xerostomia; BD—burning, dysgeusia; BXD—burning, xerostomia, dysgeusia.

**Table 2:** Comparison of comorbidities between four subgroups of BMS patients.

Parameter	B N = 19	BX N = 15	BD N = 14	BXD N = 23	*p* *
Hypertension (N, %)	7 (36.8)	5 (33.3)	6 (42.9)	10 (43.5)	0.915
Hypercholesterolemia (N, %)	1 (5.3)	1 (6.7)	1 (7.1)	2 (8.7)	0.979
Cardiovascular diseases (N, %)	3 (15.9)	1 (6.7)	1 (7.1)	1 (4.3)	0.620
Respiratory diseases (N, %)	0	0	2 (14.3)	2 (8.6)	0.309
Gastrointestinal diseases (N, %)	11 (57.9)	5 (33.3)	7 (50.0)	12 (52.2)	0.537
Endocrine diseases (N, %)	1 (5.3)	1 (6.7)	1 (7.1)	2 (8.7)	0.979
Hypothyroidism (N, %)	4 (21.1)	2 (13.3)	2 (14.3)	2 (8.7)	0.724
Hyperthyroidism (N, %)	1 (5.3)	1 (6.7)	0	0	0.512

All data are given as whole numbers (percentage) or arithmetic mean ± standard deviation. * chi-square test. Abbreviations: BMS—burning mouth syndrome; B—burning; BX—burning, xerostomia; BD—burning, dysgeusia; BXD—burning, xerostomia, dysgeusia.

**Table 3:** Comparison of medication between four subgroups of BMS patients.

Parameter	B N = 19	BX N = 15	BD N = 14	BXD N = 23	*p* *
Calcium Channel Blockers (N, %)	6 (31.6)	4 (26.7)	4 (28.6)	6 (26.1)	0.981
Thiazide Diuretics (N, %)	1 (5.3)	1 (6.7)	1 (7.1)	5 (21.7)	0.286
Beta blockers (N, %)	4 (21.1)	2 (13.3)	3 (21.4)	3 (13.0)	0.845
Antiplatelets (N, %)	3 (15.8)	0	2 (14.3)	2 (8.7)	0.434
Anticoagulants (N, %)	1 (5.3)	0	0	0	0.427
Bisphosphonates (N, %)	1 (5.3)	2 (13.3)	1 (7.1)	1 (4.3)	0.740
Levothyroxine (N, %)	3 (15.8)	2 (13.3)	1 (7.1)	3 (13.0)	0.905
Statins (N, %)	3 (15.8)	1 (6.7)	2 (14.3)	2 (8.7)	0.804
Proton Pump Inhibitors (N, %)	9 (47.4)	3 (20.0)	3 (21.4)	7 (30.4)	0.280
Analgesics (N, %)	2 (10.5)	6 (40.0)	3 (21.4)	4 (17.4)	0.196
Allopurinol (N, %)	0	1 (6.7)	0	1 (4.3)	0.850

All data are given as whole numbers (percentage) or arithmetic mean ± standard deviation. * chi-square test. Abbreviations: BMS—burning mouth syndrome; B—burning; BX—burning, xerostomia; BD—burning, dysgeusia; BXD—burning, xerostomia, dysgeusia.

**Table 4:** Comparison of burning characteristics between four subgroups of BMS patients.

Parameter	B N = 19	BX N = 15	BD N = 14	BXD N = 23	*p* *
Worst in the morning (N, %)	4 (21.1)	2 (13.3)	1 (7.1)	3 (13.0)	0.717 *
Worst in the evening (N, %)	7 (36.8)	4 (26.7)	10 (71.4)	13 (56.5)	0.059 *
Same morning/evening (N, %)	8 (42.1)	8 (53.3)	3 (21.4)	7 (30.4)	0.281 *
Continuous (N, %)	17 (89.5)	12 (80.0)	8 (57.1)	17 (73.9)	0.185 *
Improvement during eating (N, %)	14 (73.7)	11 (73.3)	12 (85.7)	17 (73.9)	0.826 *

All data are given as whole numbers (percentage) or arithmetic mean ± standard deviation. * chi-square test. Abbreviations: BMS—burning mouth syndrome; B—burning; BX—burning, xerostomia; BD—burning, dysgeusia; BXD—burning, xerostomia, dysgeusia.

**Table 5:** Comparison of the presence of subjective symptoms, symptom duration and VAS between four subgroups of BMS patients.

Parameter	B N = 19	BX N = 15	BD N = 14	BXD N = 23	*p* *
Burning (N, %)	19 (100)	15 (100)	14 (100)	23 (100)	0.989 *
Xerostomia (N, %)	0	15 (100)	0	23 (100)	<0.001 *
Dysgeusia (N, %)	0	0	14 (100)	23 (100)	<0.001 *
*Globus hystericus* (N, %)	1 (5.3)	0	0	0	0.427 *
Paresthesia (N, %)	0	0	1 (7.1)	2 (8.7)	0.402 *
Dysesthesia (N, %)	0	0	1 (7.1)	0	0.247 *
Feeling of swelling (N, %)	1 (5.3)	2 (13.3)	0	2 (8.7)	0.540 *
Feeling of hypersalivation (N, %)	1 (5.3)	0	0	1 (4.3)	0.693 *
Change in tongue morphology (N, %)	2 (10.5)	2 (13.3)	2 (14.3)	1 (4.3)	0.727 *
Symptom duration (months)	15.8 ± 21.0	15.5 ± 30.0	9.1 ± 10.1	21.8 ± 32.9	0.557
VAS	5.3 ± 1.8	6.4 ± 1.6	6.4 ± 1.5	6.1 ± 1.6	0.216 ^†^

All data are given as whole numbers (percentage) or arithmetic mean ± standard deviation. * chi-square test. † One-way analysis of variance (ANOVA) with post hoc Scheffe test. Abbreviations: BMS—burning mouth syndrome; B—burning; BX—burning, xerostomia; BD—burning, dysgeusia; BXD—burning, xerostomia, dysgeusia; VAS—visual analogue scale.

**Table 6:** Comparison of burn topography between four subgroups of BMS patients.

Parameter	B N = 19	BX N = 15	BD N = 14	BXD N = 23	*p* *
Generalized (N, %)	4 (21.1)	2 (13.3)	3 (21.4)	3 (13.0)	0.845
Gingiva (N, %)	3 (15.8)	2 (13.3)	3 (21.4)	1 (4.3)	0.460
Lips (N, %)	4 (21.1)	3 (20.0)	4 (28.6)	6 (26.1)	0.932
Cheeks (N, %)	1 (5.3)	1 (6.7)	0	1 (4.3)	0.827
Tongue (N, %)	11 (57.9)	11 (73.3)	9 (64.3)	17 (73.9)	0.675
Palate (N, %)	1 (5.3)	5 (33.3)	3 (21.4)	7 (30.4)	0.166

All data are given as whole numbers (percentage) or arithmetic mean ± standard deviation. * chi-square test. Abbreviations: BMS—burning mouth syndrome; B—burning; BX—burning, xerostomia; BD—burning, dysgeusia; BXD—burning, xerostomia, dysgeusia.

**Table 7:** Multivariable logistic regression analysis of the independent predictors for B.

Parameter	OR	95.0% CI	*p*
Age	0.979	0.937–1.024	0.368
Female *	3.802	0.856–16.884	0.049
Worst in the morning	3.45 × 10^6^	/	0.995
Worst in the evening	8.22 × 10^6^	/	0.995
Same morning/evening	5.36 × 10^6^	/	0.995
Symptom duration	1.001	0.977–1.024	0.934
VAS	0.723	0.494–1.056	0.093

* The reference group is male. Abbreviations: B—burning; OR—odds ratio; CI—confidence interval; VAS—visual analogue scale.

**Table 8:** Multivariable logistic regression analysis of the independent predictors for BX.

Parameter	OR	95.0% CI	*p*
Age	0.992	0.945–1.042	0.775
Female *	0.510	0.054–4.803	0.556
Worst in the morning	8.95 × 10^9^	/	0.995
Worst in the evening	0.000	/	0.995
Same morning/evening	0.000	/	0.995
Symptom duration	1.002	0.979–1.026	0.831
VAS	1.284	0.865–1.905	0.214

* The reference group is male. Abbreviations: BX—burning, xerostomia; OR—odds ratio; CI—confidence interval; VAS—visual analogue scale.

**Table 9:** Multivariable logistic regression analysis of the independent predictors for BD.

Parameter	OR	95.0% CI	*p*
Age	0.976	0.929–1.026	0.346
Female *	1.371	0.209–8.974	0.742
Worst in the morning	7.64 × 10^6^	/	0.993
Worst in the evening	1.57 × 10^6^	/	0.994
Same morning/evening	1.93 × 10^6^	/	0.994
Symptom duration	0.966	0.916–1.019	0.209
VAS	1.042	0.698–1.554	0.840

* The reference group is male. Abbreviations: BD—burning, dysgeusia; OR—odds ratio; CI—confidence interval; VAS—visual analogue scale.

**Table 10:** Multivariable logistic regression analysis of the independent predictors for BXD.

Parameter	OR	95.0% CI	*p*
Age	1.048	0.999–1.100	0.051
Female *	0.298	0.050–1.753	0.180
Worst in the morning	4.79 × 10^6^	/	0.995
Worst in the evening	3.64 × 10^6^	/	0.995
Same morning/evening	2.74 × 10^6^	/	0.995
Symptom duration	1.010	0.991–1.030	0.283
VAS	1.054	0.762–1.458	0.749

* The reference group is male. Abbreviations: BXD—burning, xerostomia, dysgeusia; OR—odds ratio; CI—confidence interval; VAS—visual analogue scale.

## Data Availability

The data analysed in the current study are available upon reasonable request by email to the corresponding author.

## Supplementary Materials

The following supporting information can be downloaded at: https://www.mdpi.com/article/10.3390/neurosci6020033/s1, Table S1: STROBE Statement—Checklist of items that should be included in reports of cross-sectional studies.
